# Supplementary material for: Optimizing Bioink Composition for Human Chondrocyte Expression of Lubricin
Source: Bioengineering (Basel). 2023 Aug 23;10(9):997. doi: 10.3390/bioengineering10090997 (PMC10526043; doi:10.3390/bioengineering10090997)

Figure S1: DoE design parameters

Optimal Combined design, model: quadratic by quadratic, point exchanges
Mixture components: GelMA, OMA, PBS
Numeric: crosslinking time 15-60s
Categoric: Ca
60 runs
Design Constraints

**Low Limit**		**Constraint**		**High Limit**
0.000	≤	A:GelMa	≤	12.000
86.000	≤	B:PBS	≤	98.000
0.000	≤	C:OMA	≤	2.000
2.000	≤	A + C	≤	14.000
		**A+B+C**	**=**	**100.000**

Figure S2: Lap/shear setup

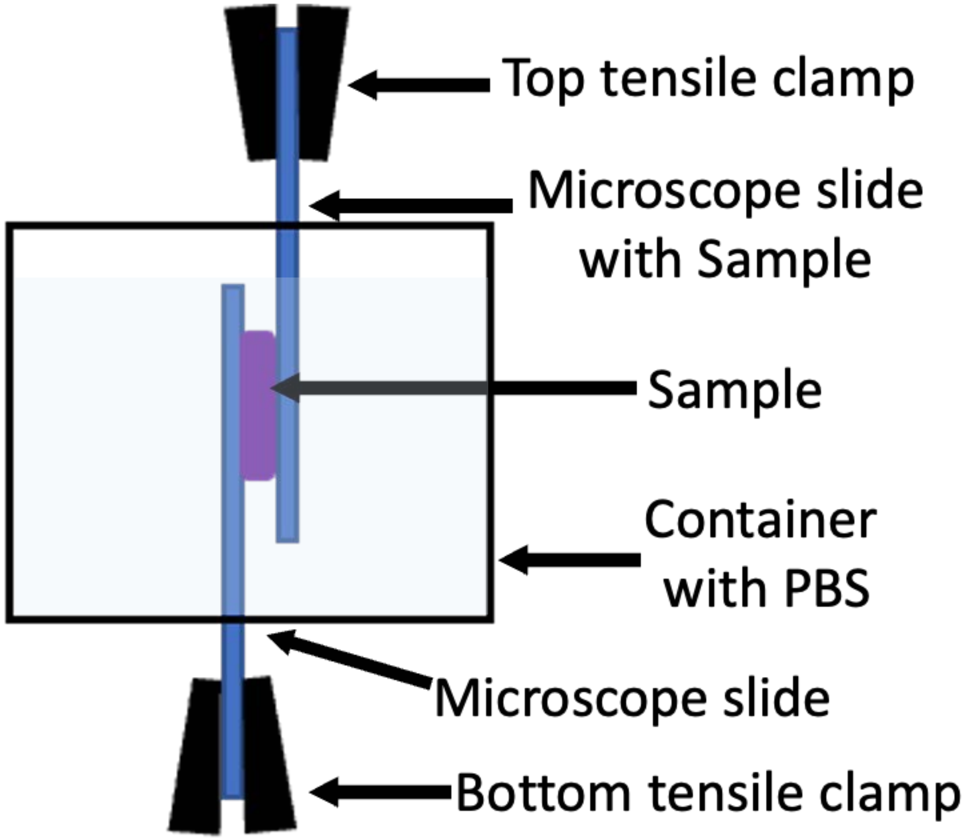

Figure S3: DoE temporal ANOVA results

Source	Term df	Error df	F-value	p-value
**Day 3**				
**Subplot**	10	41.27	5.73	< 0.0001
Linear Mixture	2	46.37	7.42	0.0016
AB	1	45.46	4.89	0.0321
AC	1	47.54	12.24	0.001
BC	1	47.45	11.64	0.0013
Bd	1	28.64	4.07	0.0532
Be	1	26.32	11.77	0.002
Ad^2	1	45.75	4.41	0.0414
Cd^2	1	48.59	4.6	0.037
ACd^2	1	46.69	13.28	0.0007
**Day 8**				
**Subplot**	5	46.81	7.36	< 0.0001
Linear Mixture	2	44.17	3.18	0.0512
AB	1	54	3.59	0.0636
BC	1	42.29	21.1	< 0.0001
Cd	1	23.46	5.54	0.0274
**Day 10**				
**Subplot**	13	33.83	4.24	0.0004
Linear Mixture	2	37.03	11.94	< 0.0001
AB	1	46	6.64	0.0132
AC	1	37.56	23.49	< 0.0001
Ad	1	39.26	0.0521	0.8207
Ae	1	42.95	0.0518	0.8211
Cd	1	43.8	0.2483	0.6208
Ce	1	45.65	0.1981	0.6584
ACd	1	45.79	0.2899	0.5929
ACe	1	44.55	0.029	0.8656
Ade	1	39.16	0.0211	0.8852
Cde	1	43.86	0.2424	0.625
ACde	1	45.86	1.03	0.3155

Terms: A = GelMA, B = PBS, C = OMA

Source	Term df	Error df	F-value	p-value
**Day 14**				
**Subplot**	5	43.96	8	< 0.0001
Linear Mixture	2	40.91	12	< 0.0001
AB	1	53.69	7.5	0.0084
AC	1	40.81	17.5	0.0001
Be	1	16.62	4.28	0.0545
**Day 16**				
**Subplot**	7	39.48	5.58	0.0002
Linear Mixture	2	41.65	1.2	0.3103
AB	1	51.81	3.36	0.0724
Ae	1	44.06	0.03	0.8632
BC	1	39.95	17.06	0.0002
Be	1	46.96	0.1142	0.7369
ABe	1	51.74	1.04	0.3125
**Day 20**				
**Subplot**	5	47.54	10.82	< 0.0001
Linear Mixture	2	42.38	3.07	0.0566
AB	1	52.24	4.07	0.0489
BC	1	41.3	27.52	< 0.0001
Ce	1	38.16	3.3	0.0771
**Day 22**				
**Subplot**	7	39.56	5.39	0.0002
Linear Mixture	2	39.61	14.02	< 0.0001
AB	1	51.99	3.25	0.077
AC	1	39.49	17.18	0.0002
Ad	1	46.81	0.0397	0.843
Bd	1	48.78	0.1341	0.7158
ABd	1	43.02	3.03	0.0888

Figure S4. DNA and GAG/DNA for validation data to support moving forward with these groups

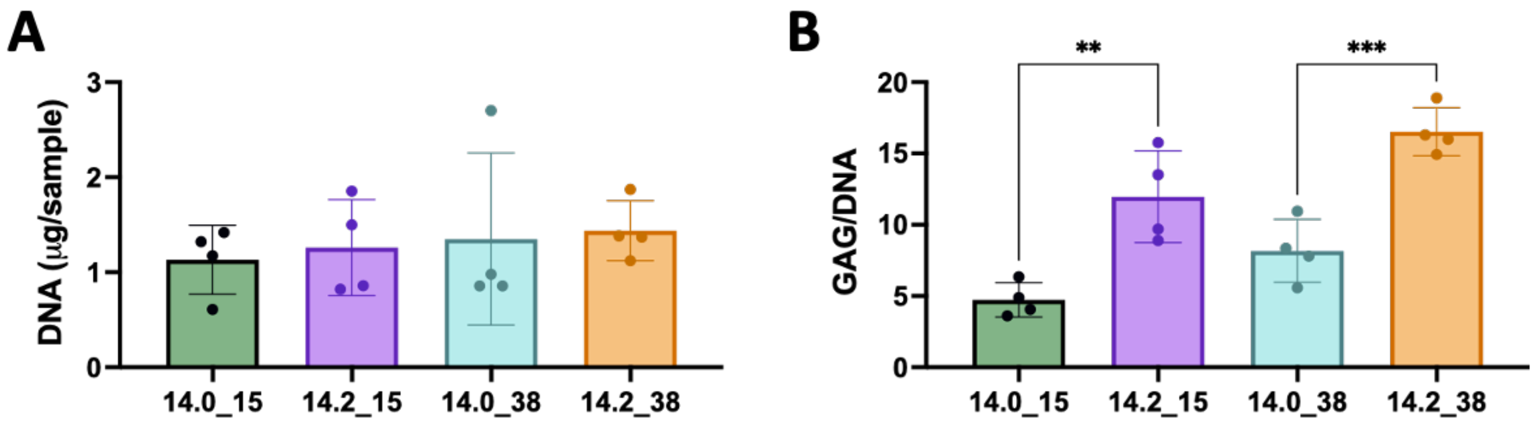

Figure S5: Chondrocyte viability in printed and pipetted hydrogels

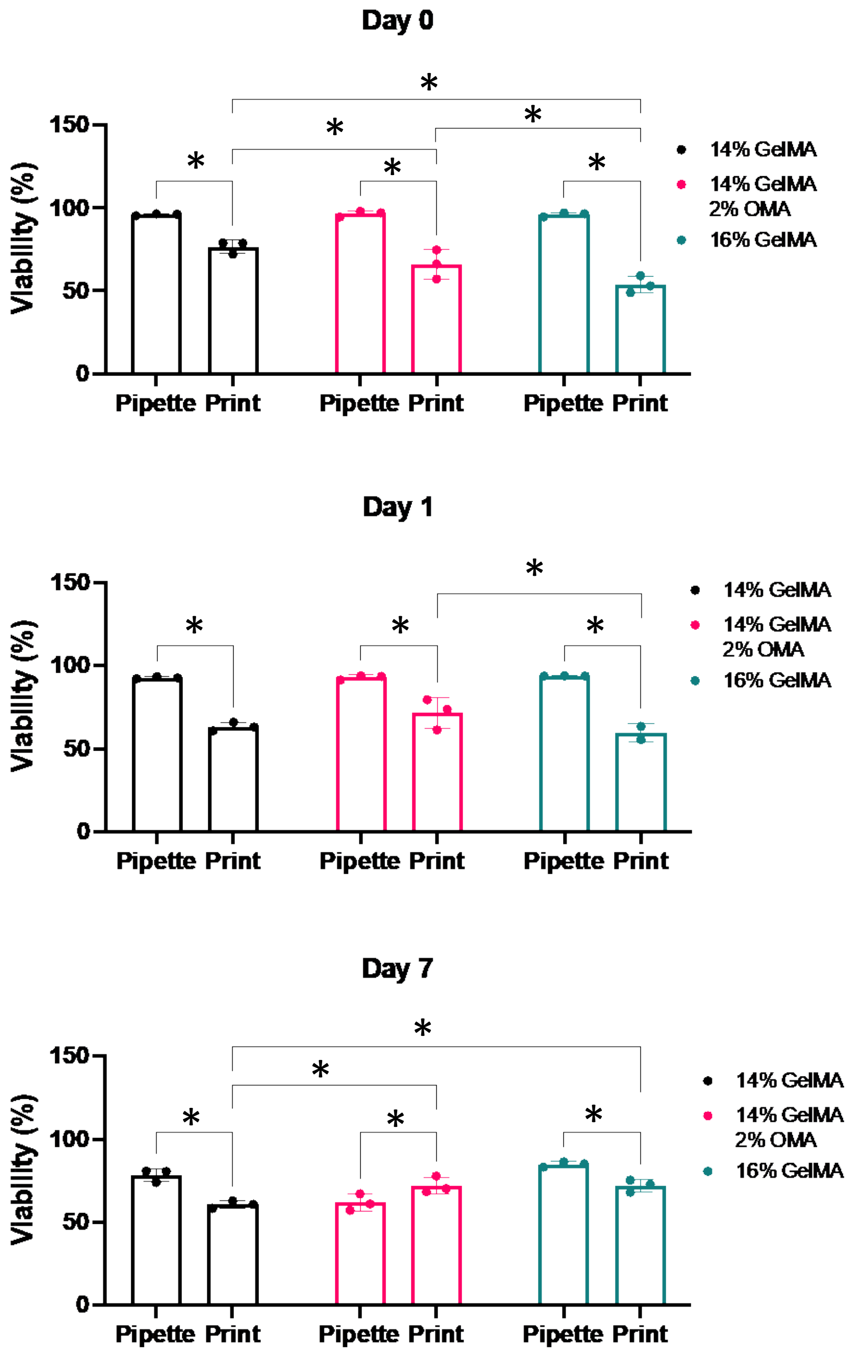

Figure S6: Cumulative lubricin driven luminescence vs. storage and loss moduli

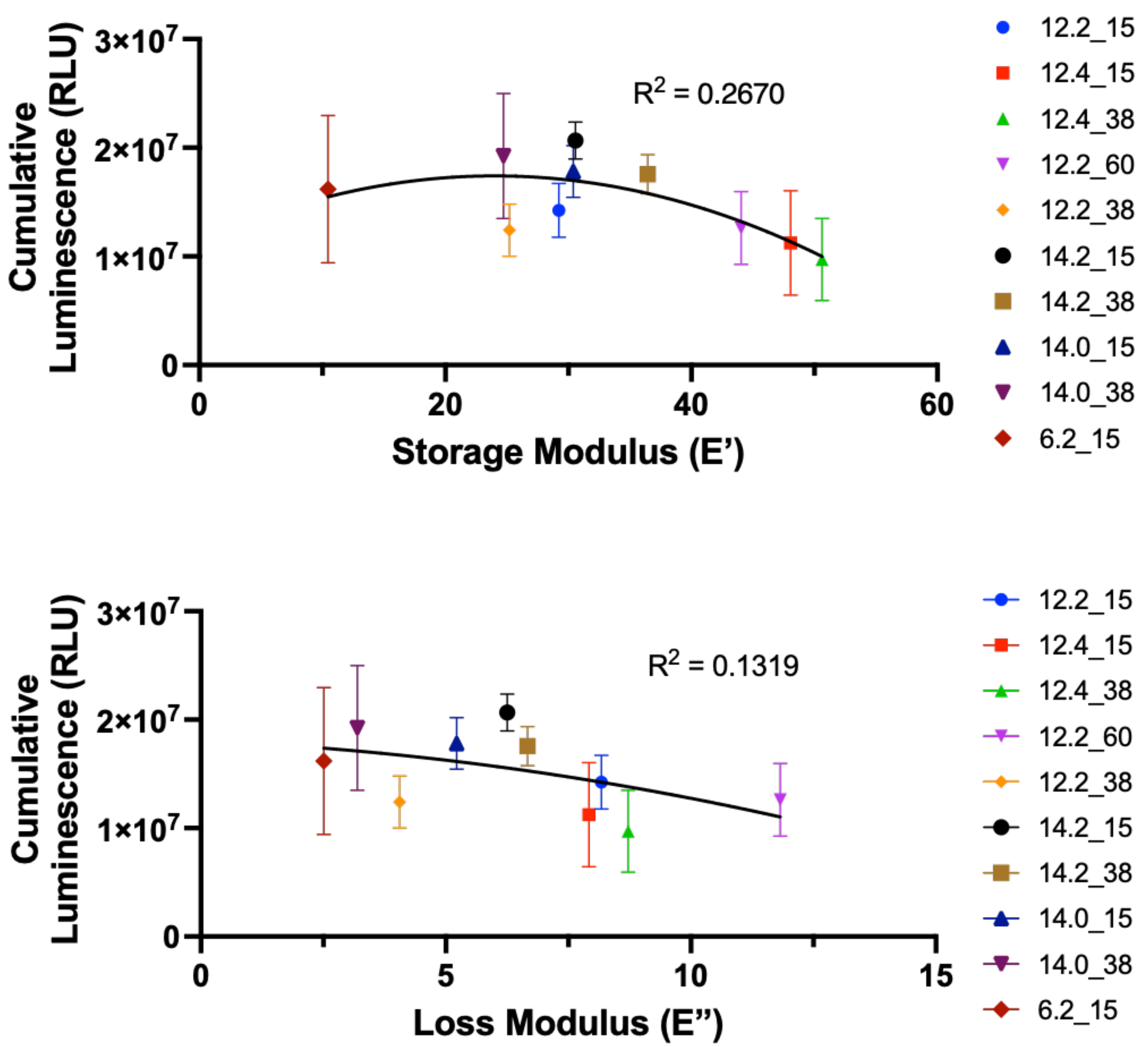

Figure S7: Loss Modulus, Tan Delta and Complex modulus at day 0 and 22

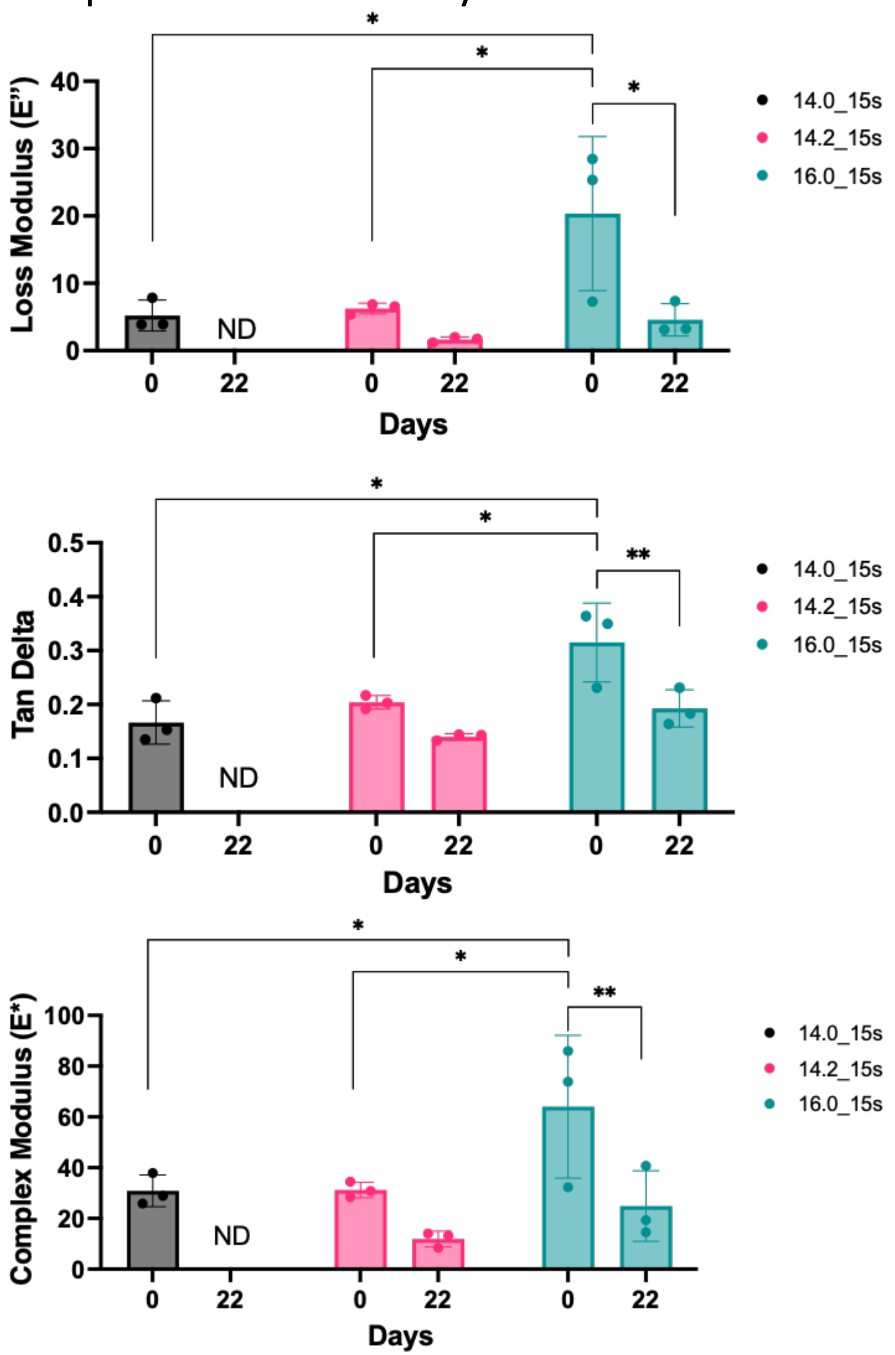

Supplement: Supplementary file 1 [file bioengineering-10-00997-s001.zip › bioengineering-2472134-supplementary.pdf]
